# Conservative non‐surgical management in medication related osteonecrosis of the jaw: A retrospective study

**DOI:** 10.1002/cre2.303

**Published:** 2020-07-02

**Authors:** Massimo Albanese, Francesca Zotti, Giorgia Capocasale, Stefano Bonetti, Fabio Lonardi, Pier Francesco Nocini

**Affiliations:** ^1^ Section of Dentistry and Maxillofacial Surgery, Department of Surgical Sciences, Paediatrics and Gynecology University of Verona Verona Italy; ^2^ independent researcher Verona Italy

**Keywords:** conservative non‐surgical therapy, MRONJ, oral diseases, osteonecrosis

## Abstract

**Objectives:**

To date, the best treatment for Medication Related Osteonecrosis of the jaw (MRONJ) is controversial. Recent studies suggest different therapies, considering the stage of MRONJ; however, sometimes patients, although with remarkable extension of disease, cannot undergo surgery. The purpose of present preliminary study was to evaluate the efficacy of conservative non‐surgical treatment of MRONJ lesions in a cohort of patients ineligible for surgery or refusing any surgical treatment for stage II and III of MRONJ.

**Materials and methods:**

Patients with MRONJ (staging II or III) ineligible for surgical treatment were selected for a retrospective study. A conservative non‐surgical therapy (antibiotics and antiseptic) was administered for 1 year. Five scheduled checks were performed to assess changes in signs and symptoms during the observational period.

**Results:**

Our observation was carried out on 12 patients. Improvement of signs and symptoms of disease were observed in population.

**Conclusion:**

This study suggests that non‐surgical treatment may be a valid therapeutic option in patients ineligible for surgery. The sample size is small, further studies should be carried out to satisfy the aim of a conservative non‐surgical treatment protocol establishment.

## 1 INTRODUCTION

Medication‐related osteonecrosis of the jaw (MRONJ) is an adverse drug reaction, characterized by progressive destruction of bone in patients who underwent to bone‐targeting agents associated with the risk of MRONJ, in the absence of a previous radiation treatment (Campisi et al., 2014; Fusco, Bedogni, Addeo, & Campisi, 2017).

To date, two main categories of drugs associated MRONJs are recognized, acting differently to bone metabolism: antiresorptive drugs (Bisphosphonates and Denosumab) and Antiangiogenic (eg *Vascular Endothelial Growth Factor inhibitors* such as Bevacizumab, *Tyrosine Kinase* inhibitors such as Sunitinib and mammalian Target of Rapamycin inhibitors such as Everolimus) (Di Fede, Panzarella, Mauceri, et al., 2018).

Etiology of MRONJ is multifactorial and pathogenesis remains unknown (Ruggiero, Saxena, Tetradis, Aghaloo, & Ioannidou, 2018); the patient's medical history, clinical examination, and radiological data are the only diagnostic and staging tools to approach and to face this condition (Di Fede et al., 2018).

Since MRONJ is a multifactorial disease, it is difficult to develop an etiological therapy; therefore, treatments can be surgical (Nisi et al., 2018), with or without Growth Factors (Borsani et al., 2018), and non‐surgical. The non‐surgical treatments include use of systemic antibiotic therapy, also associated with hyperbaric oxygen therapy (HBO) (Ceponis, Keilman, Guerry, & Freiberger, 2017), low‐level laser therapy (LLLT) (Mauceri, Panzarella, Maniscalco, et al., 2018), and topical ozone therapy (OT) (Ripamonti et al., 2012).

The surgical treatments can be divided into conservative approaches (e.g., bone debridement, sequestrectomy) or more aggressive therapy so surgical resections and jaw bone reconstruction, where necessary (Ruggiero et al., 2018).

The best treatment for MRONJ is controversial, and there is not yet an agreement about the recommended method; however, the crucial point to face is the disease's progression by using infection control means (Vescovi & Nammour, 2010).

In recent studies, authors suggest different therapies, considering the stage of MRONJ (Iorio‐Siciliano et al., 2018); however, sometimes patients with remarkable extension of disease cannot undergo surgery, for example, for pathological conditions, with high operative risk of for neoplastic diseases considerably undermining the life expectancy; in these cases, it could be indicated conservative non‐surgical therapy.

Operative risk can be approximately predicted using The American Society of Anesthesiologists (ASA) physical status classification system. Table 1 shows the latest version as approved by the ASA House of Delegates on October 15, 2014 (Doyle, Goyal, Bansal, & Garmon, 2020).

**TABLE 1:** The American Society of Anesthesiologists (ASA) physical status classification system

ASA 1	A normal healthy patient.
ASA 2	A patient with a mild systemic disease.
ASA 3	A patient with a severe systemic disease that is not life‐threatening.
ASA 4	A patient with a severe systemic disease that is a constant threat to life.
ASA 5	A moribund patient who is not expected to survive without the operation. The patient is not expected to survive beyond the next 24 hours without surgery.
ASA 6	A brain‐dead patient whose organs are being removed with the intention of transplanting them into another patient.

Therefore, for patients classified as ASA 3 or 4, surgical treatment of stage II and III of MRONJ (following SOCIETA' ITALIANA DI PATOLOGIA E MEDICINA ORALE/*SOCIETA*' *ITALIANA DI CHIRURGIA MAXILLO‐FACCILAE‐* SIPMO*/*SICMF‐ staging (Bedogni, Fusco, Agrillo, & Campisi, 2012; Campisi et al., 2014) described in Table 2 *)* could not be considered the best treatment, not only for operative risk but also because often they refused any surgical treatment for primary pathology.

**TABLE 2:** SIPMO/SICMF *staging system*

Stage I	*Focal ONJ*
	Clinical signs and symptoms: Bone exposure, sudden dental mobility, nonhealing postextraction socket, mucosal fistula, swelling, abscess formation, trismus and gross mandible deformity hypoesthesia/paraesthesia of the lips
	CT signs: Increased bone density limited to the alveolar bone region (trabecular thickening and focal osteosclerosis), with or without the following signs: Markedly thickened and sclerotic lamina dura, persisting alveolar socket and cortical disruption
	A asymptomatic
	B symptomatic
Stage II	*Diffuse ONJ*
	Clinical signs and symptoms: Same as stage I
	CT signs: Increased bone density extended to the basal bone (diffuse osteosclerosis), with or without the following signs: Prominence of the inferior alveolar nerve canal, periosteal reaction, sinusitis, sequestra formation and oro‐antral fistula
	A asymptomatic
	B symptomatic
Stage III	*Complicated ONJ*
	Same as stage 2, with one or more of the following: Clinical signs and symptoms: Extra‐oral fistula, displaced mandibular stumps and nasal leakage of fluids
	CT signs: Osteosclerosis of adjacent bones (zygoma and hard palate), pathologic mandibular fracture and osteolysis extending to the sinus floor
	A asymptomatic
	B symptomatic

Therefore, the purpose of present preliminary retrospective study was to evaluate the efficacy of conservative non‐surgical treatment of MRONJ lesions in a cohort of patients with high operative risk (ASA 3) or for refused any surgical treatment and stage II and III of MRONJ (following SIPMO/SICMF staging [Bedogni et al., 2012; Campisi et al., 2014]).

## 2 MATERIALS AND METHODS

A retrospective analysis was carried out on patients referred to the Section of Dentistry and Maxillofacial Surgery of University of Verona (Italy), during the period 2012 to 2015; patients were selected for the present study if they had (a) II and III stage MRONJ (following SIPMO/SICMF staging [Bedogni et al., 2012; Campisi et al., 2014]), (b) they refused surgical treatments or they were not eligible for them; and (c) high operative risk (ASA 2–3 [Doyle et al., 2020]). We excluded patients with pathological fracture of the jaw, who went to operation and patients suffering from allergies.

In all cases, MRONJ diagnoses and staging were made combining medical history, clinical and radiological examination performed by local multidisciplinary teams of specialists in oral medicine, oral and maxillofacial surgery and radiology (following SIPMO/SICMF staging [Bedogni et al., 2012; Campisi et al., 2014]). All patients signed informed written consent about risks and benefits of treatment proposed.

All patients were followed for 12 months and timing of checks was scheduled as follows:

t_0_ (baseline, first visit), t_1_ (1 month), t_2_ (4 months), t_3_ (7 months), t_4_ (12 months).

‐ t_0_: first visit included general health assessment, accurate general anamnesis about previous pathologies, allergies, previous surgeries, radiotherapy, and current and past drug treatments (exclusion criteria). Clinical symptoms were investigated and clinical evidences and risk factors of MRONJ (e.g., diabetes, concurrent use of steroid) were deeply assessed as suggested by present day guidelines (Campisi et al., 2014; Iorio‐Siciliano et al., 2018).

Indeed, clinical signs and symptoms evaluated (Bedogni et al., 2012; Campisi et al., 2014; Khan et al., 2015) were: bone exposure, mucosal edema, mucosal rubor, dental infection, non‐healing post extraction socket, abscess, sudden tooth mobility, halitosis, mucous fistula, cutaneous fistula, rhinosinusitis, Vincent's sign, reported pain following VAS scale.

Also, Panoramic radiograph and Computed Tomography (CT) were carried out in order to diagnose MRONJ; radiological signs evaluated (Bedogni et al., 2012; Campisi et al., 2014; Ruggiero, Fantasia, & Carlson, 2006) were: presence of bone sequestrum, diffuse osteosclerosis, bone remodeling, periosteal reaction, pathological fracture (excluded in this study), bone cortical interruption, rhinosinusitis. Descriptive analysis of radiological data collected in t_0_ was carried out. Clinical and radiological signs and symptoms were evaluated and MRONJ was staged following SIPMO/SICMF recommendations (Bedogni et al., 2012; Campisi et al., 2014) (Table 2).

A protocol of non‐surgical therapy was administered to patients according to the present day literature (Campisi et al., 2014; Montebugnoli et al., 2007; Ristow, Otto, Troeltzsch, Hohlweg‐Majert, & Pautke, 2015; Vescovi & Nammour, 2010) as follows:

- Professional dental hygiene every 4 months for 1 year,
- Chlorohexidine (0.12%) first 7 days of every month, mouthwashes two times a day for 1 year,
- Antibiotic treatment for 7 days of every month, whenever signs of infection (suppuration) or pain occurred: amoxicillin + clavulanic acid (875 mg + 125 mg) three times per day and metronidazole (500 mg) three times per day.

When gastrointestinal disease related to the prolonged antibiotic therapy was present, ciprofloxacin (500 mg) therapy two times per day for 5 days was prescribed instead of amoxicillin+ clavulanic acid (Moretti, Pelliccioni, Montebugnoli, & Marchetti, 2011).

‐ t_1_, t_2_, t_3_, t_4_: Visits at each time‐point provided complete intra and extra oral examination performed by the same surgeon. Clinical signs and symptoms evaluated in t_0_ were scored at each time‐point.

For evaluation of healing we followed Vescovi et al.(Vescovi & Nammour, 2010) classification of “clinical success”:

Stage 0: complete mucosal healing, no symptoms, and no infection signs;

Stage I: presence of bone exposure, regression of infection signs, regression of symptoms;

Stage II: presence of bone exposure with pain, infection, and swelling in the lesion area, disappearance of cutaneous fistula, maxillary sinus infection, fracture reparation;

Stage III: presence of bone exposure with pain, inflammation, secondary infection, cutaneous fistula, and pathological fracture.

Statistical analysis was performed using SPSS® Statistics 22 (IBM, Armonk, North Castle, NY), differences between and within groups at different time‐point have been tested by Kruskal‐Wallis one‐way analysis of variance test, *p* value fixed at .05.

## 3 RESULTS

For our study, we selected 12 patients (Table 3): 7 females and 5 males; the mean age was 81.5 years.

**TABLE 3:** Patients' anamnestic data

	Age (years)	Sex	ASA	Medical conditions	Related drug/s	Affected bone	MRONJ stage
1	70	M	2	Prostate cancer	Zoledronate intravenous	Maxilla	3
2	85	F	3	Osteoporosis	Alendronate per os	Mandible	2
3	95	F	3	Rheumatoid arthritis	Denosumab and Risedronate	Mandible	3
4	70	F	2	Breast cancer	Zoledronate intravenous	Mandible	2
5	84	F	2	Osteoporosis	Zoledronate and alendronate	Mandible	2
6	69	F	2	Multiple myeloma	Zoledronate intravenous	Mandible	3
7	85	F	3	Osteoporosis	Alendronate per os	Mandible	2
8	93	F	3	Osteoporosis	Alendronate per os	Mandible	2
9	78	M	2	Prostate cancer	Denosumab	Mandible	3
10	90	M	3	Prostate cancer	Zoledronate intravenous	Mandible	2
11	60	M	2	Prostate cancer	Trastuzumab	Mandible	3
12	80	M	2	Prostate cancer	Zoledronate intravenous	Mandible	2

Prostate cancer was the most common diagnosis (33.3%), followed by osteoporosis (16.6%), breast cancer (8.3%) and rheumatoid arthritis (8.3%) and multiple myeloma (8.3%). 5 patients (41.6%) used Zoledronate intravenous, 3 patients (25%) used Alendronate per os, 1 (8.3%) patients used Denosumab subcutaneous, 1 (8.3%) patient used Trastuzumab, 1 (8.3%) patient used combined treatment with Zoledronate and Alendronate, and 1 (8.3%) patient used combined treatment with Denosumab and Risedronate.

Comorbidities were: high blood pressure, history of Transient Ischemic Attack (TIA), pulmonary hypertension, history of stroke, Parkinson's disease.

Spontaneous lesions occurred in 3 (25%) cases. A history of tooth extraction at the site of necrosis was reported by 6 (50%) patients. 1 (8.3%) patient presented severe periodontal disease, in 1 (8.3%) case MRONJ occurred for perimplantitis, in 1 (8.3%) patient had incongruous dentures.

The site affected by MRONJ was the mandible in 11 (91.6%) cases and the maxilla in 1 (8.4%) case. Clinically, 6 (50%) patients had bone exposure. Edema and rubor were present in all patients (100%). 9 (75%) had mucous fistula, 4 (33.3%) presented with cutaneous fistula. Halitosis was present in 83.3% of the patients. The pain symptoms was reported by 10 patients (Avg VAS 3/10), while the 2 patients reported Vincent's sign.

Radiologic findings at t_0_ were: osteoslerosis (presented in all cases), bone sequestrum (83.3%), bone remodeling (66.6%), periosteal reaction (66.6%), rhinosinusitis (8.33%).

**FIGURE 1:**
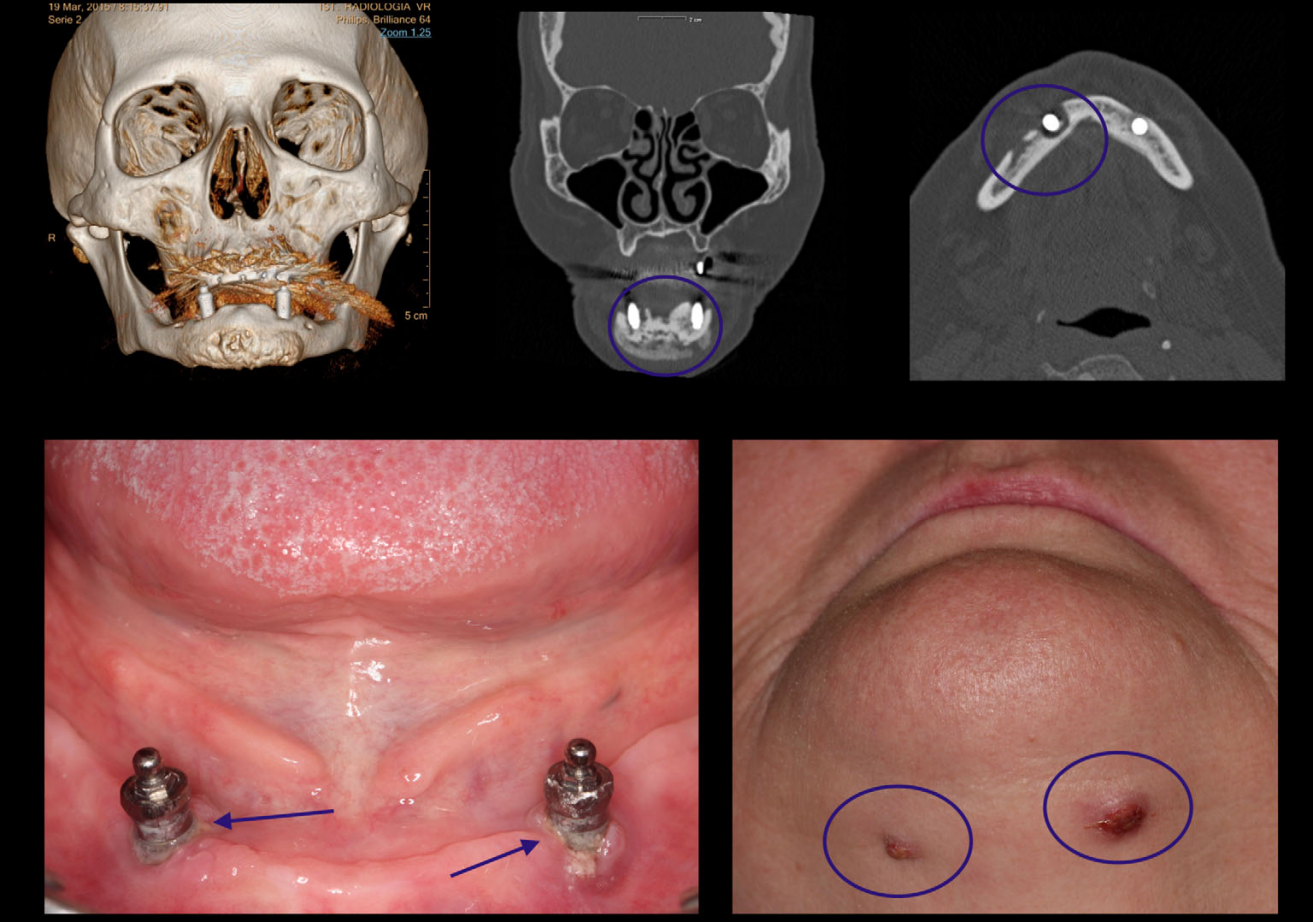
Location: Lower Jaw. Zolendronic Acid EV for more than 3 years. Peri‐implantitis, bone remodeling and cutaneous fistulas

**FIGURE 2:**
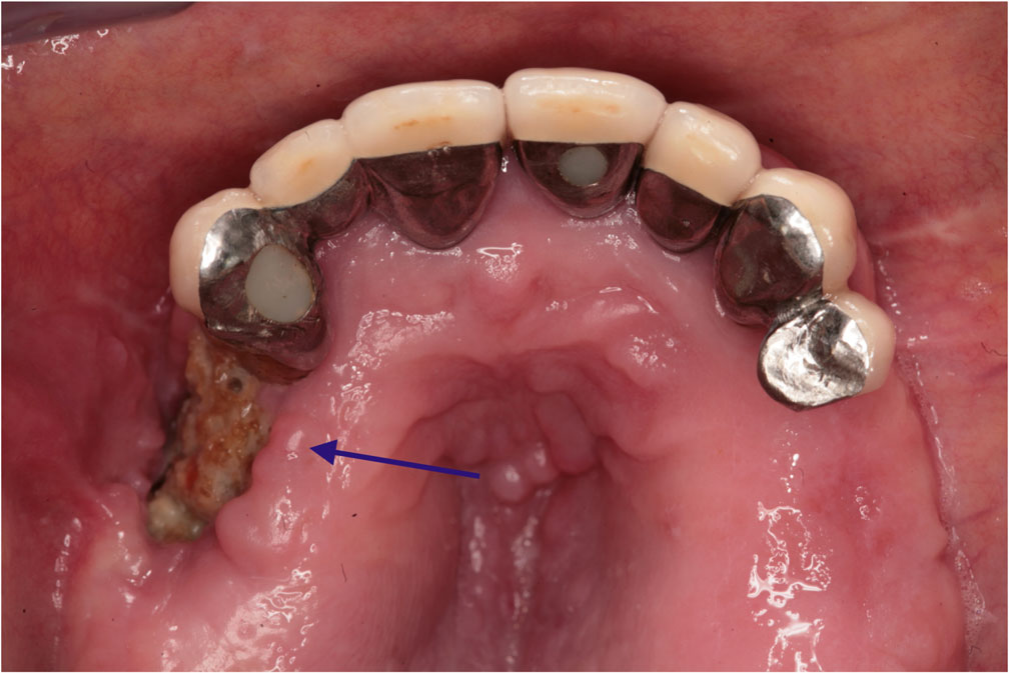
Location: Upper Jaw. Zolendronic acid for more than 3 years. Bone exposure

Some clinical and radiological images are showed in Figures 1 and 2.

MRONJ of all 12 patients was staged II and III, according to the SICMF‐SIPMO clinical and radiological staging system (Bedogni et al., 2012).

No dropout from prescriptions were reported, further no patients needed to change to ciprofloxacin. All patients were treated with only antibiotic with amoxicillin + clavulanic acid (875 mg + 125 mg) and metronidazole (500 mg) three times per day and antiseptic therapies. During the observation period, bone exposure slightly reduced and it was found to be present in 33.3% of the sample at t_4_. Differences in terms of bone exposure at different time‐points were found to be not statistically significant (*p* = .544); furthermore one patient expelled the bone sequestrum spontaneously.

Rubor and edema found to be improved: at t_2_ 16.6% reported them, whereas in t_4_ signs were completely disappeared (*p* = .00001).

Halitosis was present in 41.6% at t_1_ and in 8.3% at t_2_, no longer detectable at t_3_ and t_4_. (*p* = .00001).

Mucous fistulas progressively recovered during the observation period, found in 8.3% of the patients at t_4_. (*p* = .001); also cutaneous fistulas were lowered to 2 cases (*p* = .6567).

In one patient MRONJ was localized in upper jaw, in this case rhinosinusitis (rhinorrhea) was present at t_0_. Medical treatment completely solved this finding in t_2_. Differences within data collected at different time‐points are not statistically significant for this parameter. (*p* = .541).

Pain showed a great enhancement: it was never reported at t_4_. (*p* = .00001).

All our patients presented in t_4_ Stage 0 or Stage I following Vescovi's classification (Vescovi & Nammour, 2010).

A summary of these results were reported in Table 4.

**TABLE 4:** Trend of parameters during the observation period

Clinical examination	t_0_ (first visit)	t_1_ (1 month)	t_2_ (4 months)	t_3_ (7 months)	t_4_ (12 months)
Mucosal edema	12	12	2	1	0****
Mucosal rubor	12	12	2	2	0****
Halitosis	10	5	1	0	0****
Mucous fistula	9	8	5	1	1***
Cutaneous fistola	4	3	2	3	2*
Bone exposure	6	5	5	5	4*
Rhinosinusitis	1	1	0	0	0*
Pain Avg. (vas scale)	3	1.5	0.9	0.4	0****

*Note*: *not statistically significant. **p < .05. ***p < .001. ****p < .00001.

## 4 DISCUSSIONS

To date, the best management of MRONJ is controversial; literature promoted a stage‐dependent management of the disease (Bermúdez‐Bejarano et al., 2017; Fliefel, Tröltzsch, Kühnisch, Ehrenfeld, & Otto, 2015; Iorio‐Siciliano et al., 2018; Ristow et al., 2015; Ruggiero et al., 2014); indeed, the treatment protocol is case‐dependent, according to the condition stage and symptoms (AlDhalaan, BaQais, & Al‐Omar, 2020; Rosella et al., 2016).

In this retrospective study, we reported patients with II and III stage of MRONJ and two of these were classified ASA 2. In itself, ASA 2 does not represent a contraindication for surgical intervention, indeed the real motivation for excluding these patients was their unwillingness of undergoing a further surgery. They were affected by cancers (breast and prostate) and stage II and III of MRONJ require mild invasive surgical procedures, these issues were responsible for inclusion of ASA 2 patients conservative treatment protocol for MRONJ.

Conservative non‐surgical treatment (maintaining good oral hygiene, mouthwash, intraoral gels, analgesics, and antibiotics for discontinuing use) with clinical and radiological follow‐up was found to be usually reserved for patients in early asymptomatic stages. Moreover, a recent literature review (AlDhalaan et al., 2020) reported that conservative treatment could be implemented in patients who cannot undergo surgical treatment, but though this approach may only provide temporary clinical healing of MRONJ in 70% of the cases. The objective of this protocol is the control of infection in order to slow the disease's progression, bone necrosis progression, and pain. Indeed, it is difficult to obtain complete healing in advanced stage, thus healing of MRONJ may be defined based on clinical examination, imaging findings, or both (Beth‐Tasdogan, Mayer, Hussein, & Zolk, 2017); in detail, Vescovi et al.(Vescovi & Nammour, 2010) reported a classification of “clinical success.” Therefore, it could be considered as positive result of the treatment whether patients present Stage 0 or Stage I following Vescovi's classification.

Moreover, in this study, patients treated were suffering from other diseases (especially cancers in advanced stadium) and they were of advanced age: therefore, conservative therapy seemed to be the most suitable treatment in order to maintain a stable condition, avoiding worsening of signs and symptoms, and ensuring an acceptable quality of life.

Some authors demonstrated that non‐surgical conservative therapy may not necessarily lead to complete resolution of MRONJ, but it may symptomatically provide long‐term relief.

We are well aware about the heterogeneity of sample and we can explain this because the aim of this preliminary study was to assess the viability of this protocol in maintaining the signs and symptoms of MRONJ under control without worsening of them. In good conscience, this protocol represents for us the last one chance to ensure a better quality of life to these patients, whenever it is possible, we would look to recommend the surgical treatment.

Nevertheless, literature suggests that chlorhexidine mouthwashes and an appropriate oral hygiene may reduce mouth bacterial count, moreover reducing halitosis (Brignardello‐Petersen, 2017; Erovic Ademovski, Lingström, & Renvert, 2016). These results highlight the importance of the use of chlorohexidine mouthwashes and professional dental hygiene in a prevention and conservative non‐surgical treatment protocol for MRONJ.

In addition, it has been documented in the literature that broad‐spectrum antibiotics as amoxicillin/clavulanic acid and metronidazole are the first‐line drugs (Campisi et al., 2014).

MRONJ‐associated sinusitis usually requires a multidisciplinary treatment (Procacci et al., 2018), however, in some case, signs and symptoms may improve by using only medical treatment, such antibiotics, avoiding necessity of surgical treatment like FESS (Functional Endoscopic Sinus Surgery) (Levine & Casiano, 2017) or Caldwell‐Luc antrostomy (Datta, Viswanatha, & Shree, 2016). Although our data of rhinosinusitis remission are not statistically significant because of poor sample, we might assume that also this symptom may benefit by this conservative treatment protocol. Opportunity of avoiding surgical procedures in ineligible patients is a great chance in their management; nevertheless, a larger sample is surely advisable.

Therefore, using this protocol, satisfying results were observed in subjects affected by advanced MRONJ (stage II and III). All our patients presented in t_4_ Stage 0 or Stage I following Vescovi's classification (Vescovi & Nammour, 2010) and pain was never reported at t_4_. (*p* = .00001).

These results suggest that many symptoms and signs, such as mucosal inflammation and pain, could improve or remit with the therapy administrated. This is encouraging for patients that cannot undergo surgery, and they should be stressed during pre‐protocol talks and follow‐up to improve compliance. Furthermore, regarding such parameters, our results are highly statistically significant: this is a great opportunity to improve the living conditions of patients affected by MRONJ but non‐suitable for surgery. However, the results of this dosing regimen in the reduction of signs and symptoms of MRONJ are encouraging, especially concerning the improvement of the quality of life in palliative care.

Then, this study shows that non‐surgical treatment may be a valid option for MRONJ in patients ineligible for surgery, but sample size is small; further studies on larger samples are required to define a protocol for conservative non‐surgical treatment in MRONJ.

## AKNOWLEDGEMENTS

This work would not have been possible without the untiring support of Dr. Pasquale Procacci, distinguished researcher and talented surgeon of Department of Surgery, Dentistry, Paediatric and Gynecology of University of Verona, who have been fundamental in carrying out this research and in reaching results. We are grateful to him because he has shown us, by his example, what a good scientist, and person, should be.

The authors declare no conflict of interest. This research received no external funding.

## ETHICAL CONSIDERATIONS

All procedures performed in studies involving human participants were in accordance with the ethical standards of the institutional research committee (University of Verona, Italy) and with the 1964 Helsinki declaration and its later amendments or comparable ethical standards.

Clinical protocol was carried out, in accordance with up‐to‐date literature, with the understanding and written consent of each subject and according to the above mentioned principles.

For this study was not needed to collect an approval of IRB or ethical committee because the protocol is routinely used in our clinical practice, moreover we analyzed data and we did not perform a clnical trial neither an observational study.
